# Membrane reorganization after photochemical internalization to release transferrin-biofunctionalized polystyrene microparticles

**DOI:** 10.1038/s41598-018-35913-3

**Published:** 2018-12-04

**Authors:** Inmaculada Mora-Espí, Lleonard Barrios, Elena Ibáñez, Jorge Soriano, Carme Nogués

**Affiliations:** Unitat de Biologia Cel·lular, Departament de Biologia Cel·lular, Fisiologia i Immunologia, Facultat de Biociències, Universitat Autònoma de Barcelona, Bellaterra, 08193 Barcelona, Spain

## Abstract

Therapeutic drug carriers can drive their cargo to their target cells. However, an obstacle is usually the entrapment of the drug inside the endolysosomal compartment, which physically impedes its actuation by the impossibility of reaching its molecular site of action. To overcome this hurdle, photochemical internalization (PCI) has been proposed, but the extent of PCI-induced membrane disruption and its capability to allow the release of microparticles is unknown. The aim of the present study was to determine if PCI allows the release of microparticles from the endolysosomal compartment to the cytosol and to analyze at the ultrastructural level the effect of PCI on the membrane surrounding the particles. Confocal microscope allowed us to detect that endolysosomal membranes suffered some disruption after PCI, evidenced by the diffusion of soluble transferrin from the endolysosomes to the cytosol and by a decrease of LAMP1-microparticles co-localization. Transmission electron microscopy (TEM) showed a decrease in the number of well-defined membranes around microparticles after PCI, and scanning TEM combined with energy dispersive x-ray revealed an increase in the width of endolysosomal membranes after treatment. These results suggest that endolysosomal membranes suffered an ultrastructure alteration after PCI, enough to liberate soluble transferrin but not the entire microparticles.

## Introduction

The therapeutic effects of many drugs are limited due to several reasons, being the inefficient targeting to the desired cells and the trapping of the drug inside the endolysosomal compartment two of the most important^1,2^. Different drug carriers have been developed to direct the drugs to the target cells, such as monoclonal antibodies against specific molecules expressed only in the target cells^3^, low-density lipoproteins that interact with plasma membrane receptors^4^ or nano- and microparticles that can carry targeting molecules^5,6^as well as integrating other functions, such as biosensing^7^, cell-tagging and tracking^7–10^, or drug delivery^11–14^.

Once the drug arrives at its target cell it usually needs to be internalized to reach its molecular site of action. The plasma membrane is a natural barrier that regulates the selective entrance of molecules and particles into the cell. Large molecules and particles are normally internalized by endocytosis^15^, a process resulting in the formation of a vesicle containing the endocytosed material^16^, which ends up inside the endolysosomal compartment, usually preventing its interaction with its final molecular target.

To overcome endolysosomal entrapment, different endolysosomal escape enhancers have been proposed: (1) chemical enhancers, such as polyethylenimines (PEIs), which can buffer the endolysosomal pH by a massive accumulation of protons in their functional groups, acting as a “sponge” for protons. Proton accumulation is followed by an influx of Cl^−^ ions, causing an osmotic swelling and the endolysosomal membrane disruption that allows the release of the contents into the cytosol^17^; (2) biological enhancers, like virus and bacteria toxins, which present specific fusion proteins and cell-penetrating peptides able to mediate endolysosome scape^18^; and (3) physicochemical enhancing techniques, based on the physical principles and chemical reactions that can directly trigger the delivery of the contents of the endolysosomal compartment by its membrane disruption. Some of these techniques include the use of ultrasounds^19^, magnetic fields^20^, plasmonic nanobubbles^21^, laser photothermal treatments^22^ or photochemical internalization^23,24^.

Photochemical Internalization (PCI), developed by Berg *et al*., is based on Photodynamic Therapy^23,24^. In this approach, endolysosomal membranes are disrupted when exposed to light of a particular wavelength that excites a photosensitizer (PS) previously integrated in the membranes. In the presence of oxygen, the excited PS induces the production of reactive oxygen species (ROS), which ultimately cause the disruption of the endolysosomal membranes and the release of the organelle contents into the cytosol^25^. It has been described that using PCI therapeutic drugs^26,27^, DNA molecules for gene therapy^28^ or even nanoparticles^29^ are able to escape from the endolysosomal system^23,25,30^. Thus, PCI is a promising technique in the field of nanomedicine since it can potentiate therapeutic drug effects^23,25,26,31^. Nonetheless, due to the lack of systematic studies on the mechanisms of endolysosomal membrane disruption, it is not yet clear how macromolecules can actually escape from the endolysosomal compartment after PCI. In one of these few studies, Ohtsuki *et al*. reported that the photoinduced endosomal disruption started with a ROS-induced membrane destabilization that allowed protons to flow to the cytosol, raising the pH of the endosome. This led to the endosomal membrane disruption, although the exact mechanism remains unclear^25^.

Importantly, hitherto only molecules and nanoparticles have been reported to be able to escape from the disrupted endolysosomal vesicles^29^.Thus, given the great potential of microparticles in biomedical applications, the aim of the present study was to assess whether PCI can also allow the release of microparticles and, more specifically, how endolysosomal membranes surrounding the microparticles are affected at the ultrastructural level. To this end, microparticles of 1 and 3 µm in diameter were biofunctionalized with transferrin and targeted to SKBR3 cells, which overexpress transferrin receptors. To check the microparticles fate, lysosome-associated membrane protein 1 (LAMP1) immunofluorescence was performed and evaluated under a confocal laser scanning microscope (CLSM). Finally, to evaluate the ultrastructural alteration of the endolysosomal membranes after PCI, transmission electron microscopy (TEM) and scanning TEM (STEM) combined with energy dispersive x-ray (EDX) analysis were used.

## Material and Methods

### Cell cultures

The breast epithelial cancer cell line SKBR3 (ATCC, Manassas, VA), which overexpresses transferrin receptors in the plasma membrane, was used in this work. Cells were cultured in McCoy’s 5 A modified medium (Gibco, Paisley, UK) supplemented with 10% fetal bovine serum (Gibco) and maintained at 37 °C and 5% CO_2_. Unless specified, all the experiments were performed 24 h after seeding.

### Biofunctionalization of polystyrene microparticles

Carboxylated polystyrene microparticles of 1 or 3 µm (µP-1 or µP-3) in diameter (Polybead® Carboxylated Microspheres. Polysciences Inc, Warrington, PA) were biofunctionalized with Transferrin from human serum-Alexa Fluor® 488 conjugate (Tf-A488. Life Technologies, Carlsbad, CA) using the PolyLink Protein Coupling Kit for COOH Microspheres (Polysciences) and according to manufacturer’s instructions.

The success of the biofunctionalization was evaluated in two ways. First, biofunctionalized microparticles were examined under a fluorescence inverted microscope (Olympus IX71, Olympus, Hamburg, Germany) to detect green fluorescence. Second, changes in zeta potential were also used to corroborate Tf-A488 biofunctionalization. The biofunctionalized microparticles were resuspended in serum-free culture medium, sonicated for 5 min (Fisherbrand FB15047, Fisher Scientific, Germany) to achieve a monodisperse suspension and their zeta potential was measured using a Zetasizer Nano ZS (Malvern Instruments, Malvern, UK). The results of the Tf-A488 biofunctionalized microparticles (µP-Tf-A488) were compared with those obtained for non-biofunctionalized microparticles in the same conditions. All the measurements were carried out in triplicate.

For the experiments, the biofunctionalized µP-1 or µP-3 were sonicated for 5 min before their incubation with cells in serum-free medium. To preserve the ratio between microparticles surface and cells, µP-1 (3.14 μm^2^) or µP-3 (28.27 μm^2^) were added in a proportion of 45 or 5 microparticles/cell, respectively.

### Photochemical Internalization

Al (III) Phthalocyanine chloride disulfonic acid (AlPc. Frontier Scientific Inc, Logan, UT) was used as PS at different concentrations (0, 1 and 2 µg/ml). The photodynamic excitation was performed by 30 s of irradiation with red light, in the range of 620–630 nm, with a mean intensity of 55 mW/cm^2^ (1.65 × 10^4^ J/m^2^) (PhotoActivation Universal Light device, GenIUL, Barcelona, Spain). A negative control not exposed to irradiation was always performed as a dark toxicity (DT) control.

To optimize experimental conditions, PCI was first evaluated on cells without microparticles using a solution of Tf-A488. Cells were seeded in 35 mm diameter dishes (Nalge Nunc Int, Roskilde, Denmark) at a density of 3 × 10^5^ cells/dish. The next day, cells were incubated for 20 h in serum-free medium with 20 µg/ml Tf-A488 and 0, 1 or 2 µg/ml AlPc. Next, cells were washed thrice with phosphate-buffered saline (PBS) and incubated for 4 h in a fresh medium. Then, cells were irradiated and observed either immediately (0 min) or 7 min after irradiation under a fluorescence microscope (470–495 nm excitation and 510–550 nm emission wavelength) (Olympus IX71). Images obtained at these two time-points were used to quantify the number of discrete green dots, corresponding to endosomes or lysosomes with entrapped Tf-A488. The number of discrete green dots was divided between the total number of cells to normalize the result. The number of green dots at time 0 min was considered as the 100% of intact endolysosomes and used to calculate the reduction in the percentage of discrete green dots at 7 min in each replicate.

In experiments with microparticles, cells were seeded in 24-well dishes with coverslips at a density of 50,000 cells/well. Three days after seeding, cells were incubated with 2 µg/ml AlPc and either µP-1-Tf-A488 or µP-3-Tf-A488 in a serum-free medium for 3 h at 37 °C. Then, cells were washed thrice with PBS and incubated in fresh medium for 4 h before irradiation. After irradiation, cells were cultured for 1 h before further processing. Cells without µP-Tf-A488 and with/without AlPc were used as controls.

The cytotoxicity of the microparticles and AlPc in dark conditions and after irradiation was evaluated and compared with control cultures in absence of microparticles and AlPc in dark conditions or after irradiation. Cells were seeded in 24-well dishes at a density of 50,000 cells/well, incubated with or without µP-1-Tf-488 and with or without 2 µg/ml AlPc and irradiated for 30 s or kept in dark conditions. After 24 and 72 h of irradiation, cell viability was determined by the MTT (3-(4,5-dimethylthiazol-2-yl)-2,5-diphenyltetrazolium bromide) assay (Sigma-Aldrich, St Louis, MO), reading the 540 nm absorbance using a Victor 3 Multilabel Plate Reader (PerkinElmer, Waltham, MA).

All photodynamic experiments were performed in triplicate.

### Internalization quantification and Immunofluorescence detection of LAMP1

Cells incubated with microparticles were fixed for 15 min with 4% paraformaldehyde (PFA. Sigma-Aldrich) in PBS, washed three times with PBS, permeabilized with 0.1% Triton X-100 (Sigma-Aldrich) in PBS for 10 min, washed again with PBS (x3) and blocked with 5% bovine serum albumin (BSA. Sigma-Aldrich) for 40 min. Next, cells were incubated for 1 h with mouse anti-LAMP1 monoclonal antibody (1:250. BD Biosciences, Franklin Lakes, NJ) at room temperature. Then, cells were washed thrice with PBS and incubated for 1 h at room temperature with goat anti-mouse IgG2a Alexa Fluor® 647 conjugate secondary antibody (1:150. Life Technologies) and Phalloidin Alexa Fluor® 594 conjugate (1:80. Life Technologies) to detect the actin cytocortex. Finally, cells were washed three times with PBS, mounted in ProLong Gold (Life Technologies) and analyzed under a CLSM (Olympus IX81. Olympus, Tokyo, Japan). Three replicates were done for each condition and a minimum of 115 cells per replicate were evaluated to assess the microparticle-cell interaction and the influence of irradiation on the disappearance of the LAMP1 positive signal around the internalized microparticles.

### Sample preparation for TEM

Cells incubated with µP-3-Tf-A488 were fixed for 25 min in 2% PFA and 2.5% glutaraldehyde (EM grade, Merck, Darmstadt, Germany) in 0.1 M cacodylate buffer (Sigma-Aldrich). They were post-fixed with 1% osmium tetroxide (TAAB Laboratories Equipment Ltd, Aldermaston, England) for 2 h and dehydrated in a graded ethanol series (15 min in 30%, 30 min in 50%, 30 min in 90%, 30 min in 95% and twice 30 min in 100%), before embedding the samples in Eponate 12 resin (TED Pella Inc., Redding, USA). Polymerization was performed at 60 °C for 48 h. Ultrathin sections of 70 nm were obtained using a Leica ultracut microtome (Leica Microsystems, Wetzlar, Germany) and placed on 200 mesh copper grids. Finally, samples were contrasted with a 2% uranyl acetate solution (Sigma-Aldrich) for 30 min and subsequently with a Reynolds lead citrate solution for 5 min and observed under a TEM Jeol JEM-1400 (Jeol Ltd, Tokyo, Japan).

### STEM high angle annular dark field (HAADF) and EDX analyses

Cells incubated with µP-3-Tf-A488 were processed as for conventional TEM experiments. However, in this case, ultrathin sections were placed on carbon-coated titanium or gold grids and were not contrasted. Cells with internalized microparticles were located and images were captured using HAADF-STEM [FEI Tecnai G2 F20 microscope operated at 200 kV and equipped with an EDX super ultra-thin window X-ray detector (FEI, Hillsboro, OR)]. For each microparticle, four equidistant transects were analyzed, acquiring STEM-EDX osmium (Os) content line profiles. Each transect was oriented from inside the microparticle to the cytoplasm, perpendicularly to with microparticle edge. Since Os tends to interact with biological membranes, an increase in the number of Os counts (i.e., intensity of the Os peak in the EDX spectrum; see Supplementary Fig. S3), indicates the presence of a lipid bilayer. For each transect analyzed, the region corresponding to the particle showed virtually no Os counts compared with the rest of the transect and was considered as background. On the other hand, in the cytoplasmic region the Os signal was usually somewhat higher than the background. To compute the width of the Os peak taking into account these two different Os level signals (particle and cytoplasm), the mean value of Os counts in the cytoplasmic region was subtracted in this region to level off the signal in the particle and cytoplasmic regions. Subsequently, the remaining Os peak was fitted to a Lorentzian curve (Supplementary Fig. S4). The width of the membrane was determined as full-width at half-maximum of the peak.

In order to obtain an average width of the membrane for the non-irradiated and irradiated cells, four transects for each particle (N = 8 for each experimental condition) were analyzed. The results for each condition were grouped in intervals of 5 nm and the corresponding distributions were fitted to a log-norm function.

### Statistical analysis

Normality was checked by the Kolmogorov-Smirnov test with the Lilliefors correction, and homogeneity of variances with the Levene test. The Chi-square test was used for LAMP1 immunofluorescence and for TEM evaluation results comparisons. The ANOVA test with Tukey’s multiple comparison test was used for PCI optimization and cytotoxicity evaluation results. The values with p < 0.05 were considered statistically significant.

## Results

### PCI-mediated release of soluble transferrin from the endolysosomal compartment

**Figure 1:**
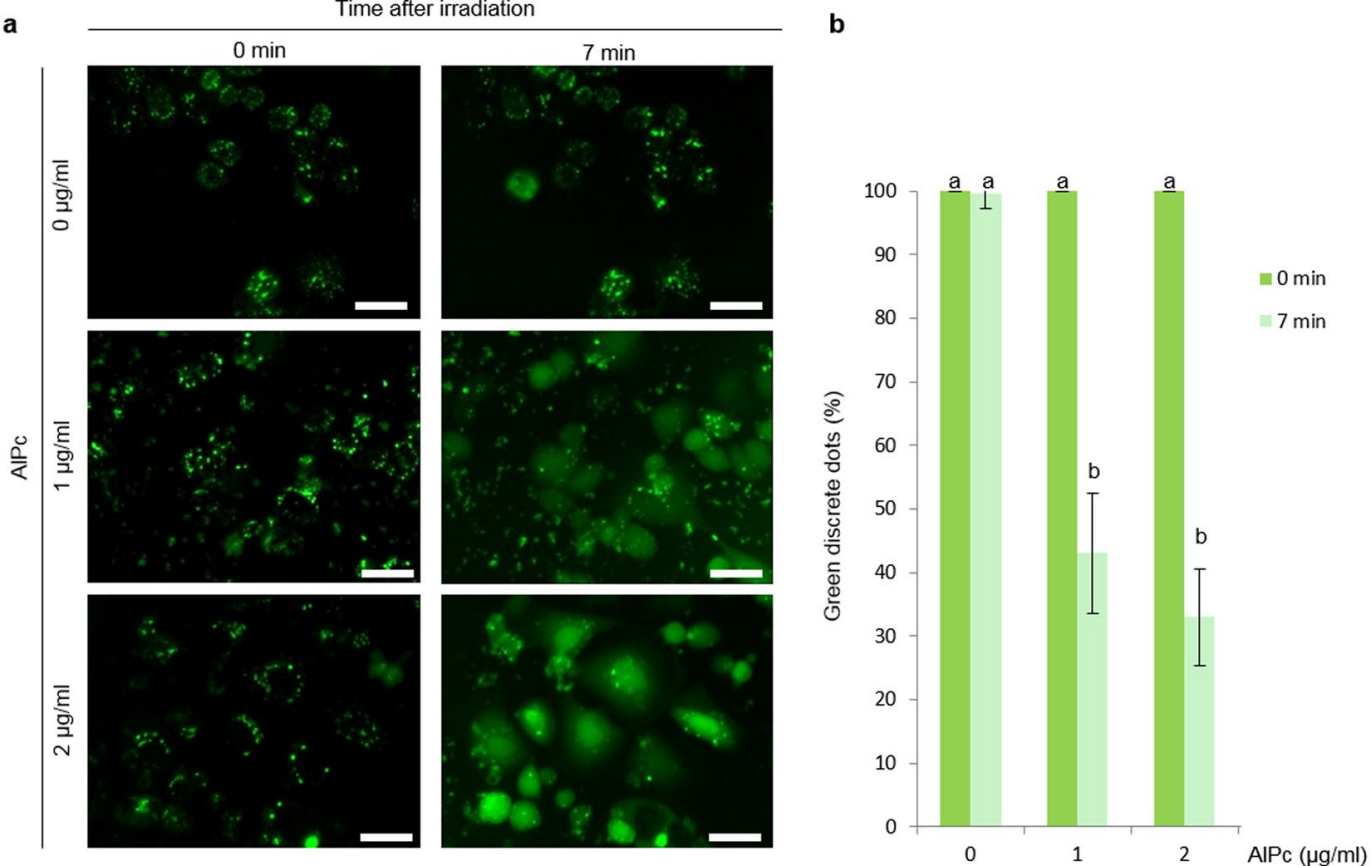
Transferrin-A488 release by photochemical internalization. (**a**) Images of cells incubated with 20 µg/ml Tf-A488 and different concentrations of AlPc (0, 1 and 2 µg/ml) captured at 0 or 7 min after irradiation with a 620–630 nm wavelength. Discrete green dots correspond to Tf-A488 entrapped inside endosomes or lysosomes. Scale bar: 30 µm. (**b**) Normalized percentage of discrete dots at 0 or 7 min after irradiation. Different letters on top of the columns denote significant differences between the two time-points for each AlPc concentration.

Regarding PCI optimization, cells incubated with 20 µg/ml Tf-A488 showed green discrete fluorescent dots under the fluorescence microscope when illuminated with 470–495 nm wavelength corresponding to endosomes or lysosomes containing Tf-A488, regardless of the AlPc concentration used (Fig. 1a). In cells without AlPc, the number of discrete green dots was maintained after irradiation. By contrast, green fluorescence rapidly diffused to the cytosol after irradiation of cells incubated with AlPc (Fig. 1a and Supplementary Movies 1–3), resulting in a significant reduction in the number of discrete green dots at 7 min after irradiation (Fig. 1b). No significant differences were detected between the two AlPc concentrations used, and the highest concentration of AlPc tested (2 µg /ml) was selected to perform the subsequent experiments.

### Microparticles biofunctionalization and internalization

**Figure 2:**
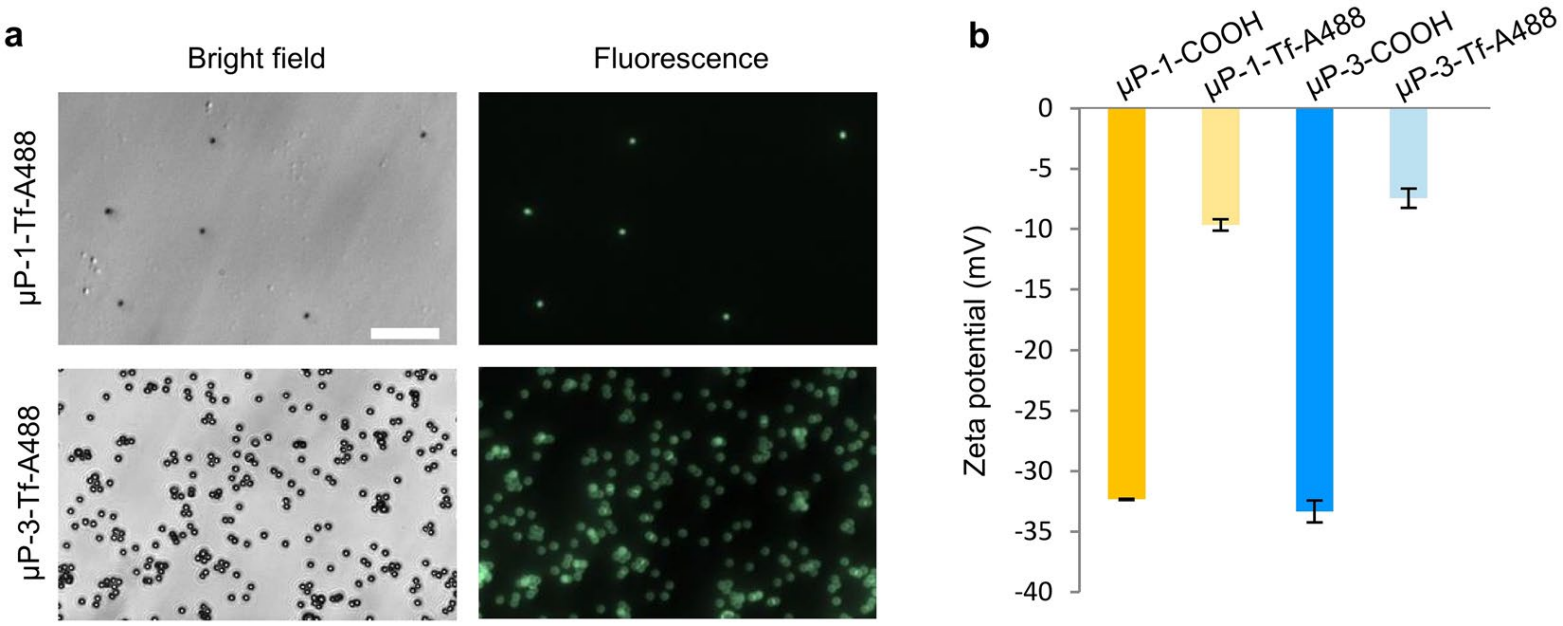
Microparticles biofunctionalization with transferrin-A488. (**a**) Images of biofunctionalized µP-1-Tf-A488 and µP-3-Tf-A488 under bright field (left) and fluorescence (right). Scale bar: 30 µm. (**b**) Zeta potential of carboxylated (COOH) and biofunctionalized (Tf-A488) microparticles.

Microparticles biofuctionalized with Tf-A488 showed green fluorescence (Fig. 2a). Biofunctionalization also produced electrochemical changes in the microparticles surface, as detected by the decrease in the negativity of the Zeta potential (Fig. 2b). Non-biofunctionalized microparticles showed highly negative Zeta potential values (−32.3 mV for µP-1 and −33.3 mV for P-3) that decreased to less negative values (between −9.7 mV and −7.4 mV) after biofunctionalization.

**Figure 3:**
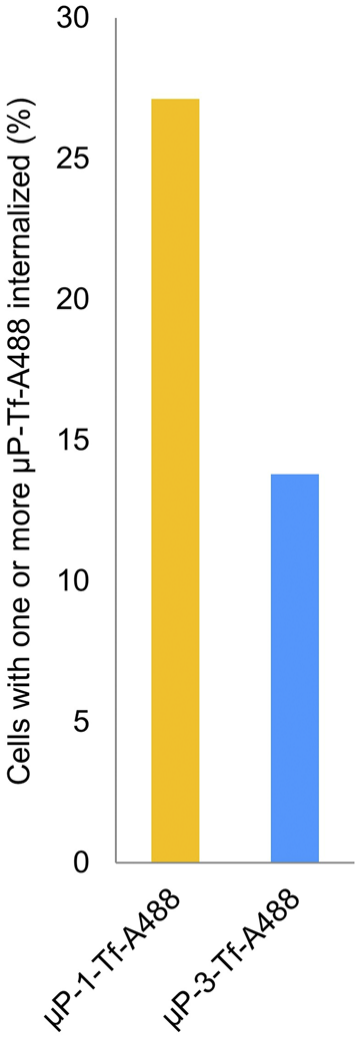
µP-Tf-A488 internalization. Percentage of SKBR3 cells with internalized µP-Tf-A488 particles (1 or 3 µm).

When the biofunctionalized microparticles were added to the cell cultures, 27.1% of cells internalized one or more µP-1-Tf-A488. However, the microparticle intake decreased to 13.8% for µP-3-Tf-A488 (Fig. 3).

### Cytotoxicity evaluation of the microparticles and the PCI treatment

Cell viability was not significantly affected by the presence of microparticles and/or AlPc, neither on the cells irradiated nor on the ones maintained in dark conditions. This lack of cytotoxicity was observed at both 24 and 72 h after irradiation (Supplementary Fig. S1).

### Endolysosomal membrane disruption after microparticles internalization and PCI treatment

**Figure 4:**
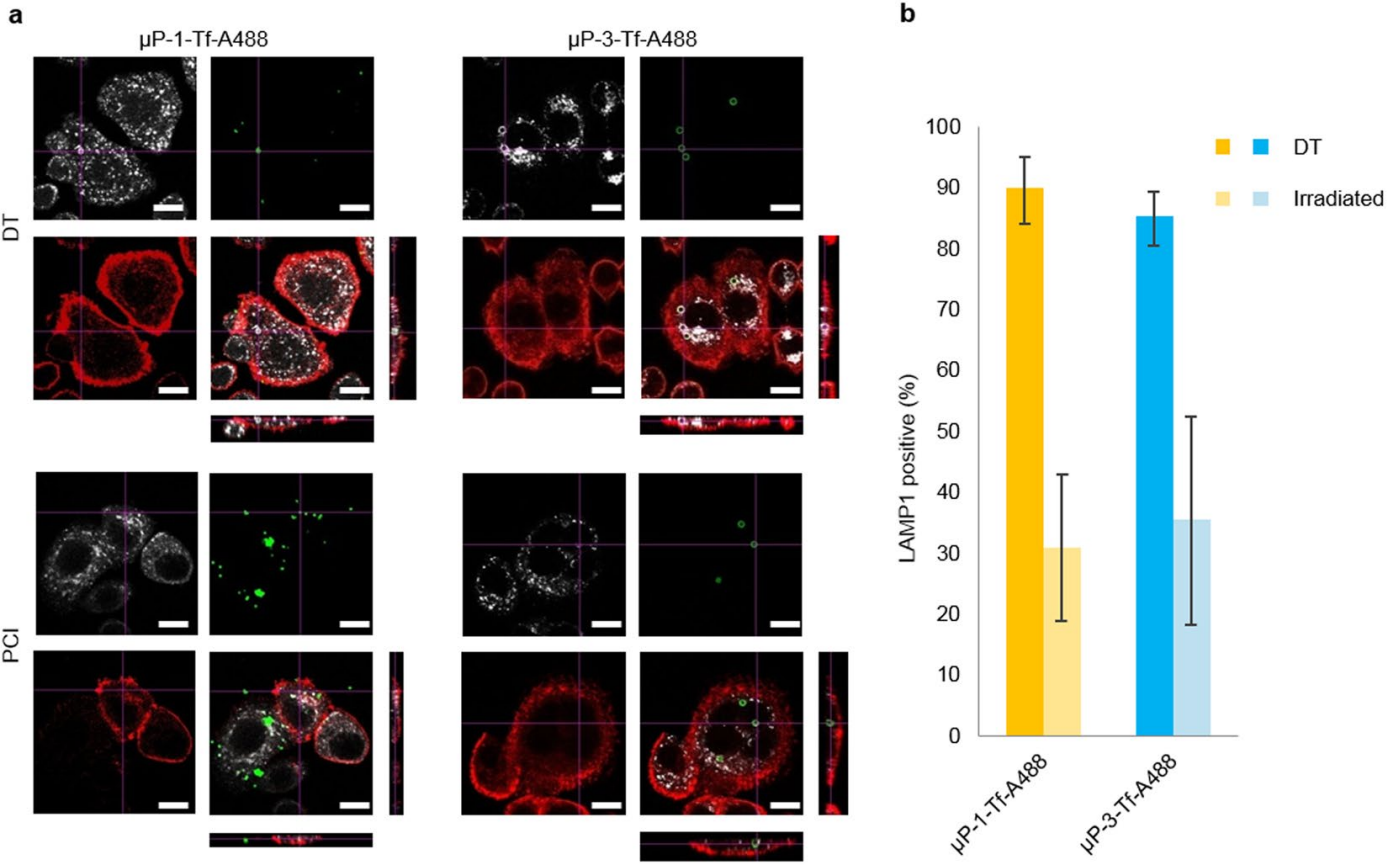
µP-Tf-A488, LAMP1 and actin co-localization analysis. (**a**) Orthogonal images of control (DT) and irradiated (PCI) samples incubated with µP-1-Tf-A488 or µP-3-Tf-A488 and 2 µg/ml AlPc. LAMP1 signal appears in white, µP-Tf-A488 in green and actin in red. Scale bar: 15 µm. (**b**) Percentage of µP-Tf-A488 co-localizing with the LAMP1 signal.

Cells incubated with biofunctionalized microparticles and AlPc were irradiated and, after 1 h, subjected to different techniques to assess the integrity of the membrane surrounding the internalized microparticles. First, LAMP1 immunodetection was used to score the number of internalized microparticles surrounded by endolysosomal membranes (Fig. 4a). In DT conditions, most of the internalized µP-1-Tf-A488 (90.0%) and µP-3-Tf-A488 (85.4%) were clearly surrounded by a positive LAMP1 signal, indicating that the presence of an endolysosomal membrane around the microparticles. Nevertheless, after irradiation, the co-localization of LAMP1 signal with µP-1-Tf-A488 and µP-3-Tf-A488 significantly decreased to 31.0% and 35.7%, respectively (Fig. 4b). No statistical differences were found between µP-1 vs µP-3 neither in DT conditions nor after irradiation.

**Figure 5:**
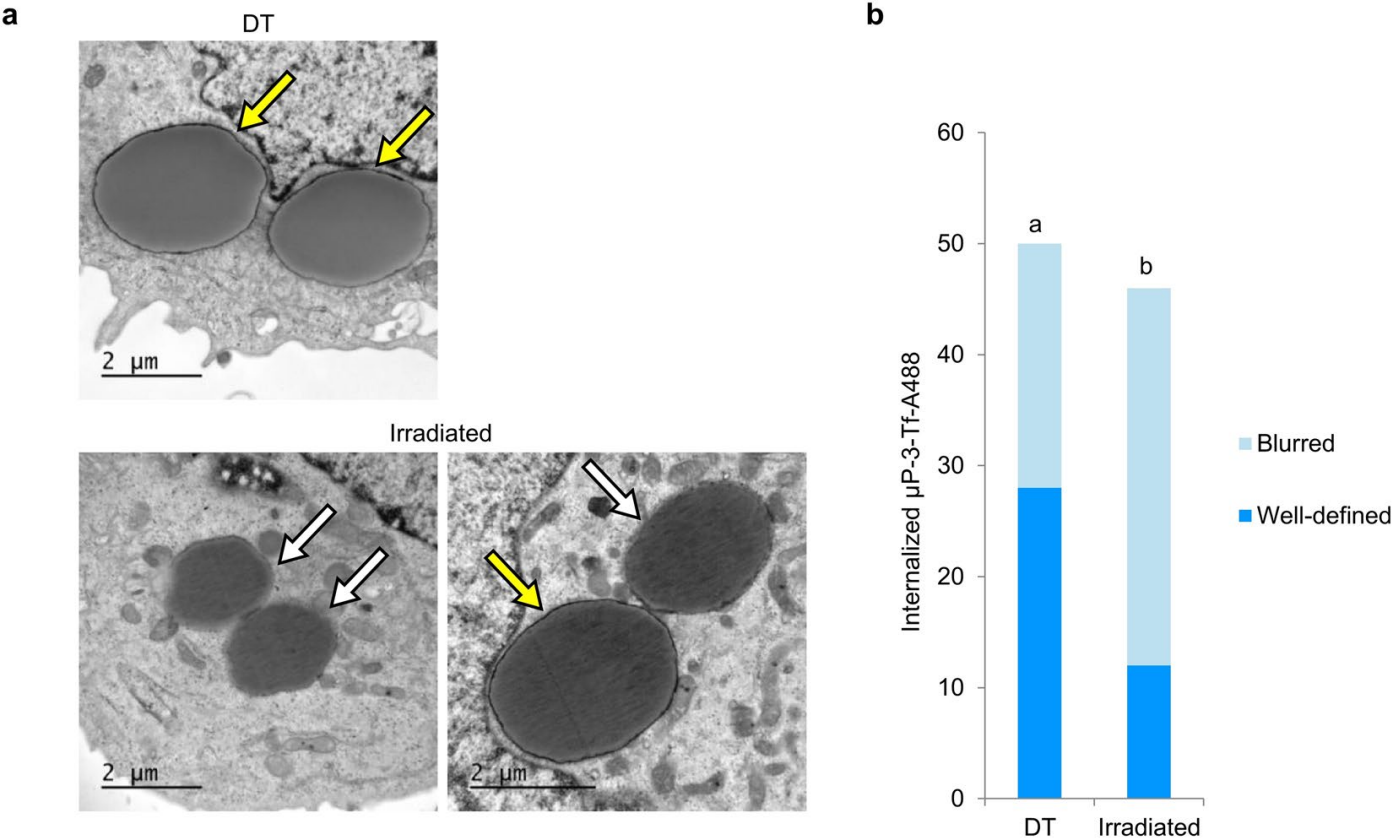
TEM evaluation of membranes surrounding internalized µP-3-Tf-A488. (**a**) Internalized µP-3-Tf-A488 in non-irradiated cells (DT) or in irradiated cells incubated with 2 µg/ml AlPc with well-defined (yellow arrows) or blurred (white arrows) surrounding membranes. (**b**) Number of internalized µP-3-Tf-A488 in control (DT) or irradiated cells surrounded by either well-defined or blurred membranes. Different letters on top of the columns denote significant differences between the distributions of the two types of membranes.

In addition, ultrastructural studies were performed in the cells incubated with µP-3-Tf-A488. In particular, a TEM analysis of the integrity of the membranes surrounding the microparticles was performed on 50 and 46 internalized µP-3-Tf-A488 in DT and irradiated cells, respectively (Fig. 5a). The number of microparticles surrounded by either a “well-defined” or a “blurred” membrane was recorded. As shown in Fig. 5b, the number of internalized microparticles surrounded by well-defined membranes was significantly higher in the DT samples than in the irradiated ones.

**Figure 6:**
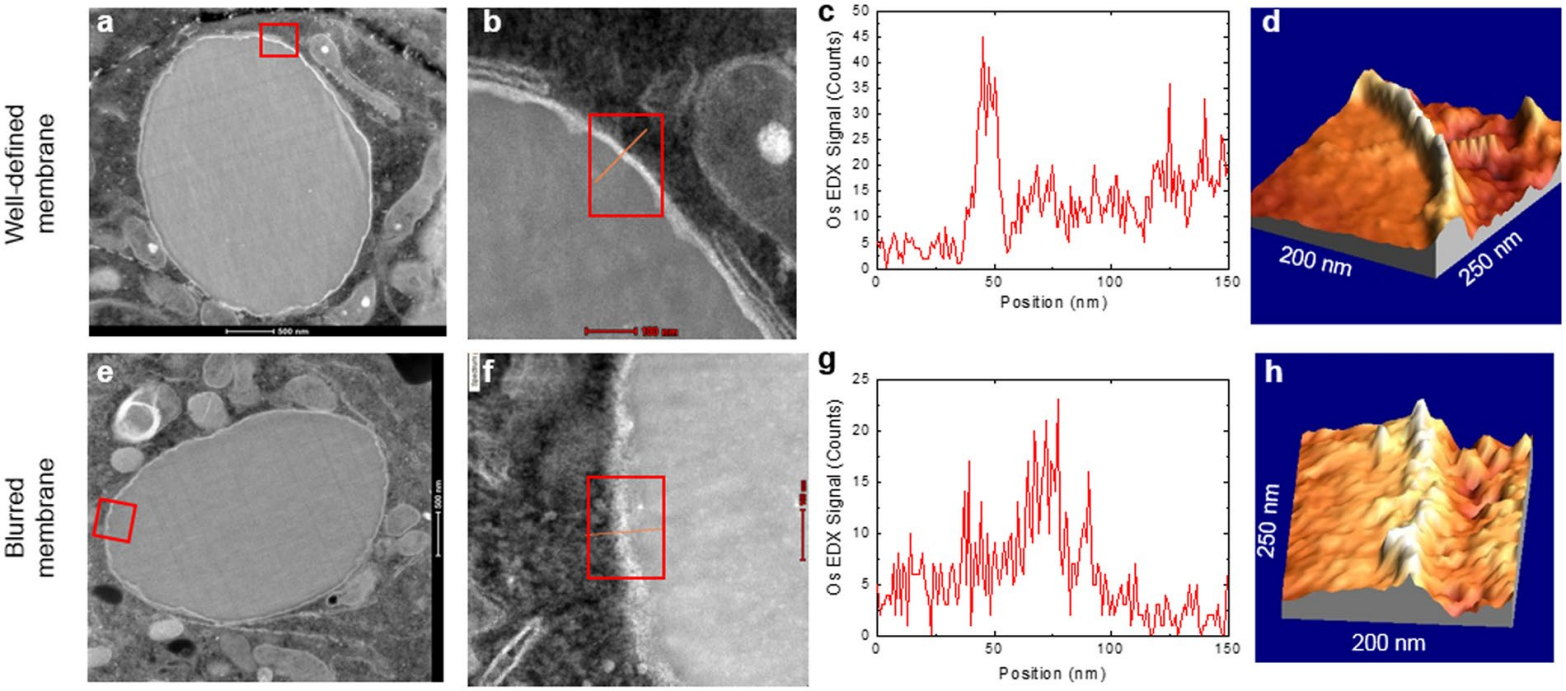
Membrane disruption evaluation by STEM HAADF-EDX. (**a**,**e**) Image of a microparticle inside an endolysosome. (**b**,**f**) Detail of the membrane surrounding the microparticle of the areas highlighted in (**a**,**e**) with the transect (line) and area (rectangle) analyzed. (**c**,**g**) Os EDX-counts profile. (**d**,**h**) 3D image of the membrane. The upper panel corresponds to a microparticle surrounded by an intact membrane (DT), with a clear Os EDX-peak and a well-defined 3D image of the membrane. The bottom panel corresponds to a blurred membrane (irradiated), with a considerably broader Os EDX-peak and a 3D image of the membrane.

Finally, HAADF-STEM was used to confirm LAMP1 immunodetection and TEM results, as in the STEM images (Fig. 6) membranes appear more distinct than in conventional TEM (Fig. 5). This is due to the large atomic number of Os (Z = 76) and the imaging technique in HAADF-STEM, which is mostly sensitive to Z^2^. In fact, well-defined and blurred membranes could be easily distinguished in STEM images (Fig. 6b,f). Furthermore, the 3D image of the HAADF-STEM intensity also clearly evidenced the difference in membrane integrity between the well-defined (DT) and blurred (irradiated) membranes (Fig. 6d,h).

**Figure 7:**
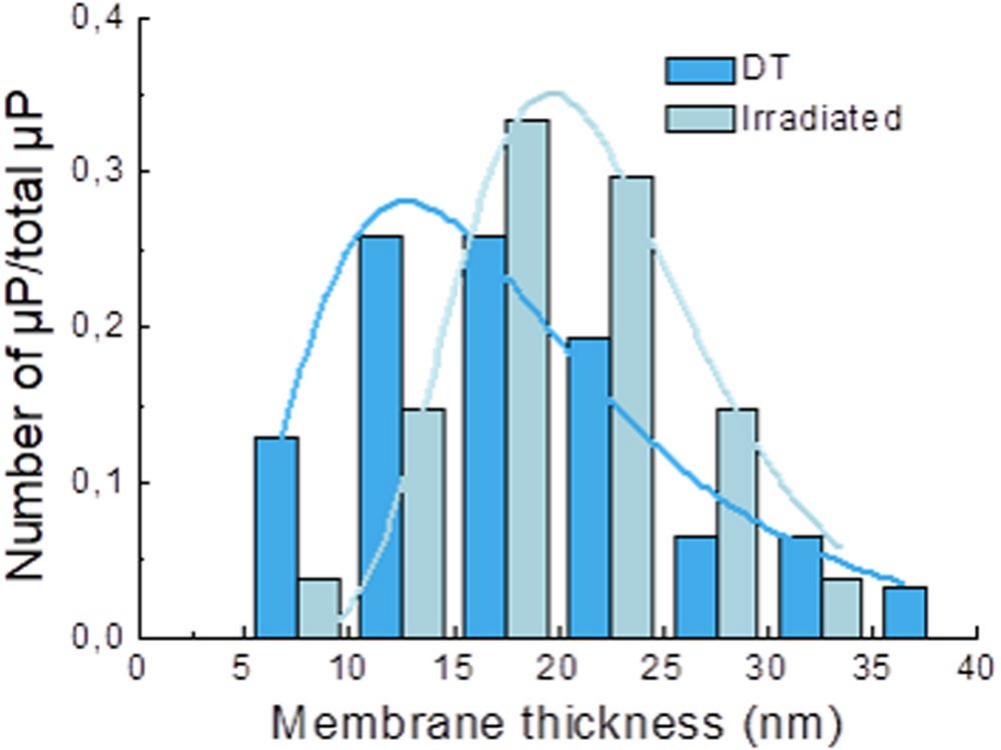
Distribution of membrane thickness for the non-irradiated (dark blue) and irradiated (light blue) samples. The lines are fits to log-normal distributions. The means of the log-normal distribution were 16.6 ± 0.5 nm for dark toxicity (DT) and 21.2 ± 0.3 nm for irradiated samples.

To quantify membrane width more accurately, STEM-EDX was used to analyze the effect of PCI by collecting Os profiles of the membranes surrounding the microparticles (Fig. 6c,g). As it can be clearly seen in Fig. 7, the width distribution of the Os peaks was shifted towards higher values (i.e., the membranes were broader) in the irradiated samples compared with the non-irradiated ones.

## Discussion

The therapeutic effects of many drugs are conditioned on their ability to recognize and enter their target cells, in order to reach their site of action and mediate their effects. However, the performance of many drugs is usually limited due to their inefficient targeting and/or endolysosomal escape. Antibody-targeted delivery of therapeutic drugs to the desired cells is a promising concept that has been developed in the last decades. For enhanced efficiency, the combination of this approach with endolysosomal release systems, such as PCI, has been proposed. Several targeting molecules have been successfully used to direct therapeutic drugs or drug carriers before performing PCI, such as a chimeric construct with vascular endothelial growth factor and gelonin for vascular targeting^33^, or antibodies against Her2 receptor^24^, epidermal growth factor receptor^34^ or EpCAM^35^ to target cancer cells. Interestingly, targeting molecules have been bound to different types of drug carriers such as polymeric micelles^36^ or nanoparticles^37^ to transport the drugs, showing enhanced therapeutic activity, reduced side effects and allowing to reduce the drug concentration due to their high specificity against the target cells^27,37^.

Compared to nanoparticles, microparticles may be advantageous for more specific targeting, since not all the cell types can internalize micrometric particles^38^. Although several reports agree that sizes between 30 and 50 nm are optimal for cell uptake^39^, it must be emphasized that nanoparticles can easily be non-specifically uptaken by non-targeted cells, leading to undesired secondary effects. In this regard, it has been reported that particles with sizes in the microscale show a higher targeting ability than those in the nanoscale^40^. In addition, the smaller nanoparticles may have increased cytotoxicity^41–43^. However, despite the potential of microparticles for biomedical purposes relatively few studies have been carried out to evaluate their internalization and posterior endolysosome release.

To analyze if PCI could assist in the release of large devices, in this study polystyrene microparticles of 1 and 3 µm in diameter were biofunctionalized with transferrin. We have previously demonstrated that the biofunctionalization of 3 µm poly-Si/Cr/Au chips with transferrin increased more than twice their internalization in SKBR3 cells, likely due to the overexpression of the TfR in their plasma membrane^44^. In the present study, we found that the biofunctionalization reduced the negative charge of the microparticles, which could further help to increase their cell-intake since it has been reported that positive or slightly negative charges facilitate endocytosis in most cell types^45,46^. In a previous work, less than 5% of SKBR3 cells were able to internalize non-functionalized µP-3 of a similar negative charge than the TF-biofunctionalized µP-3 used in the present study, which were uptaken by more than 10% of the cells. This corroborates the important role of transferrin as targeting molecule in this cell line.

Prior to performing microparticle internalization and release, the PCI technique was optimized using transferrin in solution. To be able to follow the transferrin, it was used combined with Alexa 488 (Tf-A488), similar to what other authors have done to follow the immunotoxin MOC31-gelonin during PCI^27^. Concerning the PS, Al (III) phthalocyanine chloride disulfonic acid (AlPc) was chosen for the present study due to its appropriate characteristics (e.g., hydrosolubility and amphiphilicity) for PCI^47–49^. Different concentrations of the AlPc were tested (1 and 2 µg/ml), none of which produced cytotoxic effects neither in DT nor after irradiation, in agreement with other studies^50–53^. Moreover, µP-3 have been previously described as non-cytotoxic^32^ and our results also indicate that the biofunctionalization of the microparticles with Tf-A488 does not have cytotoxic effects. To elucidate the effect of the PCI on the membranes, we first corroborated that, in dark conditions, internalized µP-Tf-A488 were indeed located inside the endolysosomal system using LAMP1 immunodetection. Interestingly, the percentage of microparticles surrounded by a LAMP1 positive signal significantly decreased after irradiation, suggesting that microparticles could have been released into the cytosol by the PCI process. Nevertheless, the remaining presence of some LAMP1 positive signal around some internalized microparticles indicates that the disruption of the endolysosomal membrane does not lead to its complete disintegration under the current conditions.

According to different reports, the PCI-induced membrane disruption, visualized as endolysosomal cargo diffusion, is due to lipid peroxidation^23,25–27,36,37,54^. To our knowledge, little research has been carried out using electron microscopy to assess the extent of membrane disruption^55,56^ and none to evaluate the ultrastructural changes resulting from PCI-induced membrane disruption. In the present study, TEM evaluation of the irradiated samples showed that membranes surrounding microparticles were frequently diffuse (probably damaged). By contrast, non-irradiated samples showed a well-defined structure of the membranes surrounding microparticles. To better understand these differences, a STEM-EDX analysis was performed. This approach allows determining the chemical elements present in a sample, and it can be used to quantify the relative abundance of a specific element at any point of a particular position of the sample under study. Despite its potential, STEM-EDX has been rarely used in cell biology. For example, it has been previously used to determine the location of specific ions in mouse induced pluripotent stem cells and mesenchymal stem cells^57^, the interaction of quantum dots with human oral epithelial cells^58^ or to locate ZnO nanoparticles in breast cancer cells^59^.Given the great potential of this methodology to track high-Z atoms, we used it to evaluate PCI-induced endolysosomal membrane disruption. The tendency of osmium (Os) to interact with biological membranes has been used for decades to fix cell membranes and to enhance their contrast for electron microscopy studies^60^. Importantly, since Os does not react with polystyrene microparticles (Supplementary Fig. S2), EDX is not only particularly suitable to determine the presence/absence of membranes surrounding microparticles, but also to infer their integrity. An increase in the Os level (Os peak) around the microparticle allows confirming the presence of a membrane and to actually measure its width. Thus, the width of the Os peak can be used as an indicator of different degree of membrane organization. In our study, the width of the Os peaks in the irradiated samples was larger than in the non-irradiated ones, which is in accordance with the images obtained in traditional TEM where membranes in irradiated samples appeared to be more diffuse. These results are also in agreement with those obtained using confocal analysis, where the absence of LAMP1 signal around microparticles in irradiated samples indicates a membrane disruption. It is important to emphasize that in all the microparticles analyzed an Os signal, although with variable width, was always present around them. Most probably, the disruption of the membrane induced by PCI corresponds to a reorganization of the lipid bilayer sufficient to release molecules in suspension such as Tf-A488 (present work), or nanoparticles^61^, but not sufficient to completely liberate a 3 µm microparticle into the cytosol. This reorganization is probably transitory because cell viability was not compromised by the PCI treatment. However, further studies are necessary to reveal this reorganization. The PCI treatment could be eventually complemented with the use of pH-sensitive bonds between the therapeutic drug and the microparticle. In the lysosomal acidic environment, these bonds would be readily cleaved, allowing the release of the drug and its escape to the cytosol due to the membrane disruption.

## Conclusions

PCI-mediated endolysosomal membrane disruption seems to consist in a partial disorganization, but not in a complete disappearance of the membrane. Ultrastructural studies showed that the endolysosomal membrane was always present around the microparticles, although showing a variable width. PCI was not sufficient to produce membrane discontinuities large enough to release microparticles. Thus, PCI could potentially be a good candidate to overcome endolysosomal entrapment of therapeutic drugs carried by microparticles only if it is complemented with the use of pH-sensitive bonds.

## Electronic supplementary material

Supplementary movie 1 (control)

Supplementary movie 2 (1 µg)

Supplementary movie 3 (2 µg)

Supplementary information
