# Supplementary material for: Membrane reorganization after photochemical internalization to release transferrin-biofunctionalized polystyrene microparticles
Source: Sci Rep. 2018 Dec 4;8:17617. doi: 10.1038/s41598-018-35913-3 (PMC6279785; doi:10.1038/s41598-018-35913-3)
Supplement: Supplementary file 4 — Supplementary information [file 41598_2018_35913_MOESM4_ESM.pdf]

Supplementary Information

Membrane reorganization after photochemical internalization to release transferrin-biofunctionalized polystyrene microparticles

Inmaculada Mora-Espí, Lleonard Barrios, Elena Ibáñez, Jorge Soriano and Carme Nogués*

Unitat de Biologia Cel·lular, Departament de Biologia Cel·lular, Fisiologia i Immunologia, Facultat de Biociències, Universitat Autònoma de Barcelona, Bellaterra, 08193 Barcelona, Spain

1. Cell viability in the presence of µP-1-Tf-A488 and AlPc, alone or combined

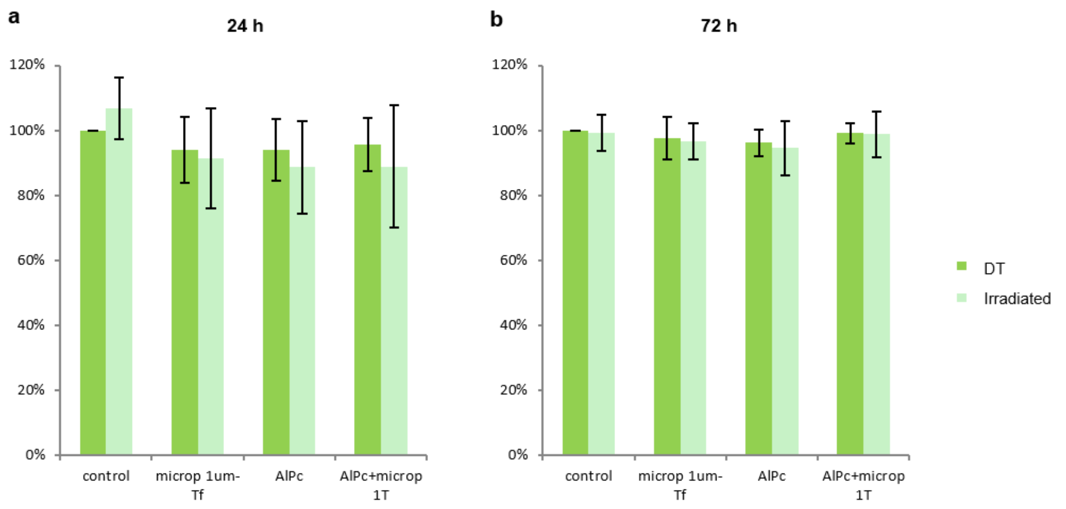

Supplementary Figure S1. Cell viability in the presence of µP-1-Tf-A488 and AlPc, alone or combined. The MTT assay was performed at 24 h (A) and 72 h (B) after irradiation or in dark conditions (DT). The cytotoxicity of microparticles and AlPc, alone and combined, was evaluated in irradiated and DT cultures. The percentage of viable cells was normalized to the DT controls.

2. EDX spectra of a free particle and an internalized one

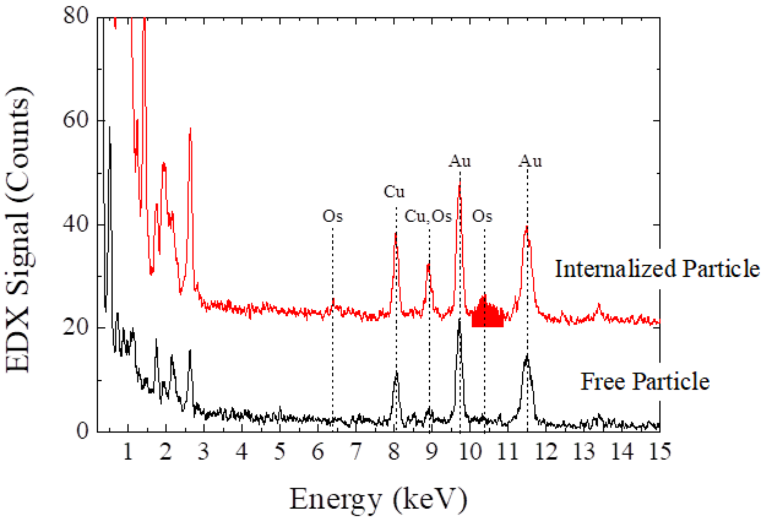

Supplementary Figure S2. EDX signal of the microparticle edge of a free microparticle (outside the cell) and an internalized one (inside an endolysosome). The Os peaks are only observed in the internalized microparticle. The peak highlighted in red was used to do the transient quantifications.

3. Correlation between the Os peaks and the presence of a lipid membrane

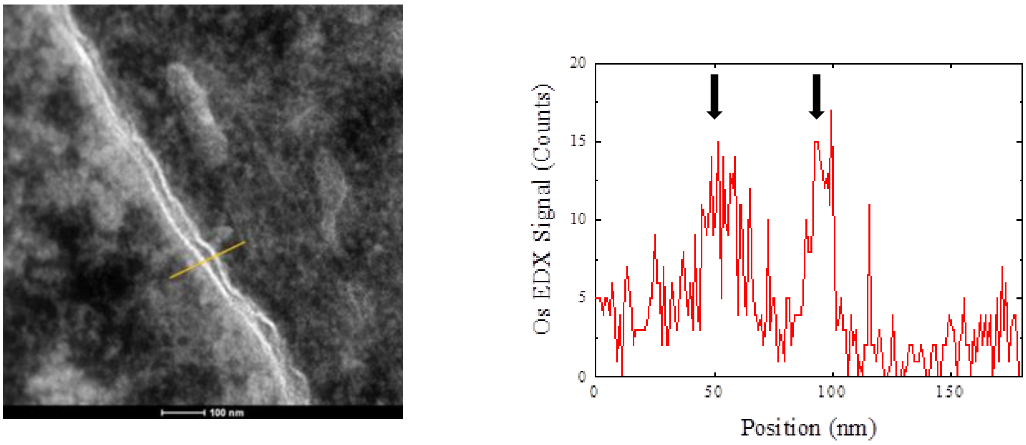

Supplementary Figure S3. (a) STEM HAADF image of a nuclear envelope showing the inner and outer membranes. The yellow line indicates the transect analyzed. (b) STEM-EDX Os content profile where two different Os peaks, corresponding to the inner and outer membrane, can be observed. N = nucleus, C = cytoplasm

4. Methodology to compute the width of the Os peak

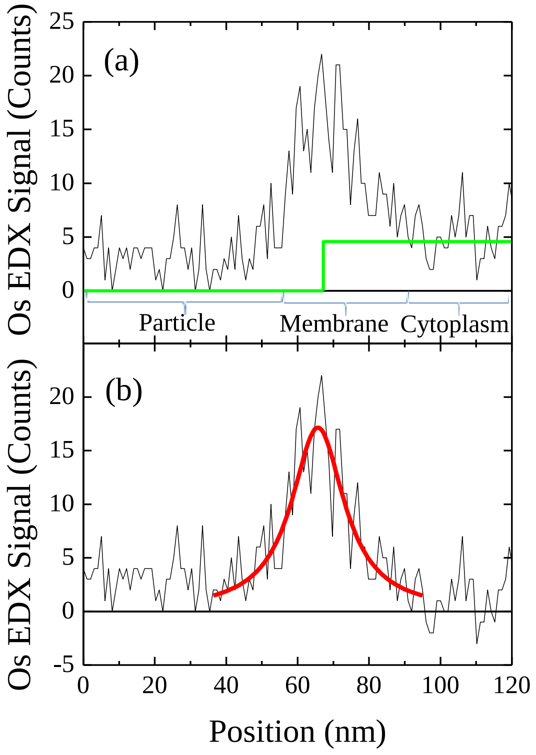

Supplementary Figures S4. (a) Os EDX signal (black) and its background signal (green). (b) Background-corrected Os EDX signal (black) with a fit to a Lorentzian function (red).

5. Release of soluble transferrin from the endolysosomal compartment

Movie 1. Control cells. No release of transferrin is observed 7 min after irradiation

https://drive.google.com/file/d/1Ld1rLYpAdJmBxz06wQSs_iIcolEzBZFP/view?usp=sharing

Movie 2. PCI treatment using AlPc (1 µg /ml). Transferrin release can be observed as the diffusion of the green fluorescence7 min after irradiation

https://drive.google.com/file/d/1HJrnBBmgqVLNCDs-6Nm0LSogi0koN5s1/view?usp=sharing

Movie 3. PCI treatment using AlPc (2 µg /ml). Transferrin release can be observed as the diffusion of the green fluorescence7 min after irradiation

https://drive.google.com/file/d/1HvNIpg85fvu5Z6anwlW7xr9jkQISSI7s/view?usp=sharing
